# Immunisation with *Neospora caninum* subunits rsNcSAG4 and rsNcGRA1 (NcSAG4 and NcGRA1 epitopes construct) in BALB/c mice: the profile of the immune response and controlling the vertical transmission

**DOI:** 10.1007/s00436-023-08020-0

**Published:** 2023-12-19

**Authors:** Cintia Fernandes Fidelis, Leandro Silva de Araújo, Pablo A. Prates-Patarroyo, Karlos H. Martins-Kalks, Leandro Licursi de Oliveira, Marlene Isabel Vargas Viloria, Gabriel A. Tafur-Gómez, Joaquín Hernán Patarroyo Salcedo

**Affiliations:** 1 Laboratório de Biologia e Controle de Hematozoários e Vetores, Departamento de Veterinária, Instituto de Biotecnologia Aplicada à Agropecuária – BIOAGRO, Universidade Federal de Viçosa – UFV, CEP, Viçosa, MG 36570-900 Brazil; 2 Laboratório de Imunoquímica e Glicobiologia, Departamento de Biologia Geral, Universidade Federal de Viçosa – UFV, CEP, Viçosa, MG 36570-900 Brazil; 3 Universidad de Ciencias Aplicadas y Ambientales – U.D.C.A, Bogotá, 111166 Colombia; 4 Patsos Biotecnologia, Parque tecnológico de Viçosa, CEP, Viçosa, MG 36570-900 Brazil

**Keywords:** *Neospora caninum*, NcGRA1, NcSAG4, Immunogen, Subunit vaccines, Recombinant peptides

## Abstract

*Neospora caninum* is an apicomplexan protozoan that causes neosporosis, which has a high economic impact on cattle herds with no available vaccine. During infection, the secretion of dense granules and the expression of surface antigens play an important role in hosting immunomodulation. However, some epitopes of those antigens are immunogenic, and using these fractions could improve the subunit antigens in vaccine design. This study evaluates the recombinant peptides rsNcGRA1 and rsNcSAG4 derived from NcGRA1 and NcSAG4 native antigens as vaccine candidates produced by a fermentative process in the yeast culture system of *Komagataella phaffii* strain Km71, confirmed by colony PCR, SDS-PAGE, and western blotting. The assay was conducted in BALB/c mice using the peptides at low (25 μg) and standard (50 μg) dosages in monovalent and combined administrations at three time points with saponin as an adjuvant assessing the immunogenicity by antibodies response and cytokine production. We challenge the females after pregnancy confirmation using 2 × 10^5^ NC-1 tachyzoites previously propagated in Vero cells. We assessed the chronic infection in dams and vertical transmission in the offspring by PCR and histopathology. Mice, especially those immunised with combined peptides and monovalent rsNcGRA1 at a standard dose, controlling the chronic infection in dams with the absence of clinical manifestations, showed an immune response with induction of IgG1, a proper balance between Th1/Th2 cytokines and reduced vertical transmission in the pups. In contrast, dams inoculated with a placebo vaccine showed clinical signs, low-scored brain lesions, augmented chronic infection with 80% positivity, 31% mortality in pups, and 81% vertical transmission. These findings indicate that rsNcGRA1 peptides in monovalent and combined with rsNCSAG4 at standard dose are potential vaccine candidates and improve the protective immune response against neosporosis in mice.

## Introduction

*Neospora caninum* is an intracellular heteroxenous protozoan that infects many animal hosts. In cattle, it is a significant cause of abortions and stillbirth (Nayeri et al. 2022). Worldwide economic losses associated with neosporosis in the cattle industry exceeded 1.3 billion USD per year, with a higher impact on dairy cattle (Reichel et al. 2013). Cows of any gestational period may abort, but most abortions occur between the fourth and sixth month of gestation (Yániz et al. 2010). During pregnancy, calves acquire the parasite by vertical transmission as the main route of infection in the exogenous and endogenous cycle (McAllister 2016). Currently, the control of neosporosis in cattle includes biosecurity strategies such as test-and-culling of seropositive cattle, reproductive management of positive herds, including the replacement of heifers, embryo transfer, and artificial insemination (Dubey and Schares 2011; Goodswen et al. 2013).

However, these strategies are expensive, and vaccination could be a cost-effective approach to managing this infection (Liu et al. 2021). A commercial vaccine for cattle neosporosis is unavailable, and the research for developing an efficient vaccine is a high priority (Monney and Hemphill 2014). The assays with live vaccines showed disadvantages, such as high production costs and the risk of causing reproductive disorders and chronic infections (Reichel and Ellis 2009). Subunit vaccines contain no live components, which improves safety. On the contrary, they contain target proteins involved in parasite cell adhesion/invasion (Hemphill et al. 2013). However, new-generation vaccines based on complete antigens exposed during natural infection showed a variable immune response due to misfolding of these or change in epitopes exposure related to the reverse vaccinology model and selecting coding genes or the recombination route which could affect the integrity of them (Goodswen et al. 2014).

The fact that *N. caninum* evades the host’s immune response makes it challenging to determine the antigen or antigen combinations in a vaccine design (Hemphill et al. 2013). The complexity of the biology of *N. caninum* suggests that an efficient subunit vaccine should include antigens spanning different stages (Fereig and Nishikawa 2020), and the effective vaccine must induce a proper immune response compatible with pregnancy and capable of preventing vertical transmission (Reichel and Ellis 2009). The focus on discovering recombinant antigens as vaccine candidates based on carefully designed antigens could offer more security, less costly production, and induce a selective immune response capable of preventing chronic infection or abortion (Monney and Hemphill 2014). Despite that, the search for recombinant antigens as a vaccine candidate remains short; some studies have focused on DNA vaccines, virus-vectorised DNA, and proteins expressed in the heterologous *Escherichia coli* system without exploration of recombinant peptides expressed in yeast (Hemphill et al. 2013; Monney and Hemphill 2014). Hence, carefully selected antigens expressed in recombinant yeast have glycosylation motifs essential to their immunogenicity. Also, antigens secreted into the extracellular medium have greater integrity and are more accessible to purification, compared to the antigens that integrate the organs of yeast (Johannssen and Lepenies 2017).

In murine models, the native antigens based on tachyzoite/bradyzoite surface and dense granule antigens (NcSAGs/NcGRAs) showed variable degrees of immune response induction against neosporosis (Monney et al. 2011). However, the immune response could be improved, including those antigens from different life cycle stages. The subunit antigens could be designed from immunogenic regions of the bradyzoite antigen NcSAG4, which is involved in the establishment of chronic infection or reactivation (Aguado-Martínez et al. 2009a), and the tachyzoite NcGRA1, which is involved in the acute infection associated with host cell penetration or tissue cyst formation (Lally et al. 1997; Vonlaufen et al. 2004). However, the reverse vaccinology on these antigens expressing their natural form induces a variable immune response because the natural form has several peptides capable of inducing a non-specific immune repertoire (Shams et al. 2022). Similarly, the coding regions of NcGRA1 used to design DNA vaccines showed little immune response with higher mortality rates after the challenge (Yu et al. 2021). Also, NcGRA1 in recombinant form failed to control the vertical transmission in pups (Ellis et al. 2008), and the NcSAG4 in recombinant form failed to generate protection against chronic infection in dams and transplacental neosporosis (Aguado-Martínez et al. 2009b). However, new-generation vaccines based on subunit peptides designed by predicting T and B epitopes from natural antigens using *in silico* tools in a reverse vaccinology approach have not been assessed against neosporosis.

The peptides based on immunogenic fractions of native antigens could improve their immunogenicity, adding new knowledge to developing new-generation vaccines against neosporosis. That could be assayed in mice which are a valid model for vaccine candidate research (Aguado-Martínez et al. 2017) due to BALB/c mice developing a productive infection with cerebral lesions (Lindsay et al. 1995; Jia et al. 2020) and vertical transmission to newborn pups after infection with *N. caninum* (Tang et al. 2023). This study evaluated in BALB/c mice potential vaccines composed of recombinant peptides rsNcGRA1 and rsNcSAG4, designed by predicting T and B epitopes of NcSAG4 and NcGRA1 antigens and produced in the yeast system of *Komagataella phaffii* concerning the immunogenicity and protection against neosporosis, demonstrating that combined peptides and rsNcGRA1 alone at standard doses can induce a proper immune response that controls the chronic infection in dams and reduces vertical transmission in pups.

## Materials and methods

### Yeast transfection

Considering the native antigens of *N. caninum* previously recognised as NcSAG4 (Fernández-García et al. 2006) and NcGRA1 (Lally et al. 1997), we predicted the immunogenic epitopes of T and B cells using IEDB analysis resources (http://tools.iedb.org/main/) and I-TASSER (https://zhanglab.ccmb.med.umich.edu/I-TASSER/) tools and determined the sequence of peptides rsNcSAG4 with 102 amino acids (10.5 kDa), and rsNcGRA1 with 84 amino acids (8.9 kDa). The synthesis of minigenes for heterologous system expression used the genomic regions involved in coding peptides but not the complete sequences previously described as NcGRA1 (GenBank: AF199030.1) and NcSAG4 (GenBank: AAW88532.1). We designed transfection cassettes inserting the synthetic minigenes encoding to rsNcSAG4 (323 bp) and the rsNcGRA1 (269 bp) peptides in the respective pPic9k vectors (650 bp) (Invitrogen USA), adding regions of His4, resistance genes for ampicillin and geneticin, and preferred codons (MATα prepro-leader*,* GenBank: AY145833) for extracellular secretion of peptides in the yeast system to complete a molecular mass of 919 bp (sNcSAG4) and 973 bp (rsNcGRA1). For *K. phaffii* strain Km 71 transfection, we linearised each vector using the restriction enzyme Sacl (Promega^®^) and introduced them inside the yeast by electroporation. We cultured the transformant yeast in selective medium (MD) without histidine (1.34% YNB, 4 × 10^−5^% biotin, 2% dextrose, 1.5% bacteriologic agar) at 30 °C until the growth of first colonies with induced the histidine (His) synthesis. We replicated these yeasts using the same MD medium with ampicillin (Sigma^®^) over five generations. We confirmed the stability of yeast cells by colony PCR using the AOX1-promoter and terminator-specific primers in the conditions published by Boettner et al. (2002). Then, we selected the colonies with high copy numbers of crossing-over genes in the YPD medium (1% yeast extract, 2% peptone, 2% dextrose, and 2% bacteriologic agar), enriched with augmented concentrations of geneticin (0.5 to 4.0 mg/mL, Sigma^®^), and these growing colonies were checked by colony PCR using the same AOX1 primers.

### Fermentative process of recombinant peptide production

For each peptide, we used the transformed colonies as mother bank inoculated separately to obtain the pre-culture batch in 70 mL of medium B (95 mM KH_2_PO_4_, 66.21 mM (NH_4_)_2_SO_4_, 37.38 mM MgSO_4_, 4.5 mM CaCl_2_.2H2O, 0.34% YPD and 4% glycerol, pH 5.0) under constant orbital agitation of 200 rpm in a shaker at 30 °C for 2 days until reaching an OD600 of 6. We microscopically confirmed the viability of the yeast of those samples of growing biomass. Each pre-culture clone grew separately by a fermentative process in a sterile bioreactor (Tec-Bio-7,5, Tecnal^®^) under positive pressure containing 4.5 L of B medium enriched with 25 mL of PTM1 trace salts and 1 mL of antifoam. Each batch fermentation grew under constant oxygen injection of 2 mmHg, agitation of 600 rpm, cooling jacket temperature of 30 °C, and pH of 5.5–6, adjusted automatically with 50% NH_4_OH or 50% H_3_PO_4_. We enriched this culture with 500 mL of 50% glycerol and 6 mL of PTM1 trace salts after reaching an OD600 of 6. We induced the yeast biomass for recombinant peptide secretion on the fourth growing day using 1% methanol over 96 h.

### Recombinant peptide filtration and confirmation

After batch fermentation, we centrifuged the yeast biomass at 7000 × *g* for 15 min at 4 °C (Thermo Scientific Multifuge X1R), recovering the supernatant containing the specific recombinant peptide. The supernatant was filtered by tangential flow filtration using the Prep/Scale Spiral-Wound Ultrafiltration Module (MilliPore^®^) and clarified using a 30 kDa filter. Less than 30 kDa permeable flow was collected and filtered using a 3 kDa filter. We desalted and sterilised this permeable with two filtration steps using a Durapore Opticap XL4 (MilliPore^®^) with 0.45 and 0.22 μm cartridges and analysed this with SDS-PAGE and western blotting. Permeable samples were recovered in sterile flasks to confirm the peptides produced (Patarroyo et al. 1995). Finally, the solution containing the recombinant peptides for vaccine formulations by bicinchoninic acid assay (BCA) was quantified (Smith et al. 1985).

### Vaccination regime

**Fig. 1:**
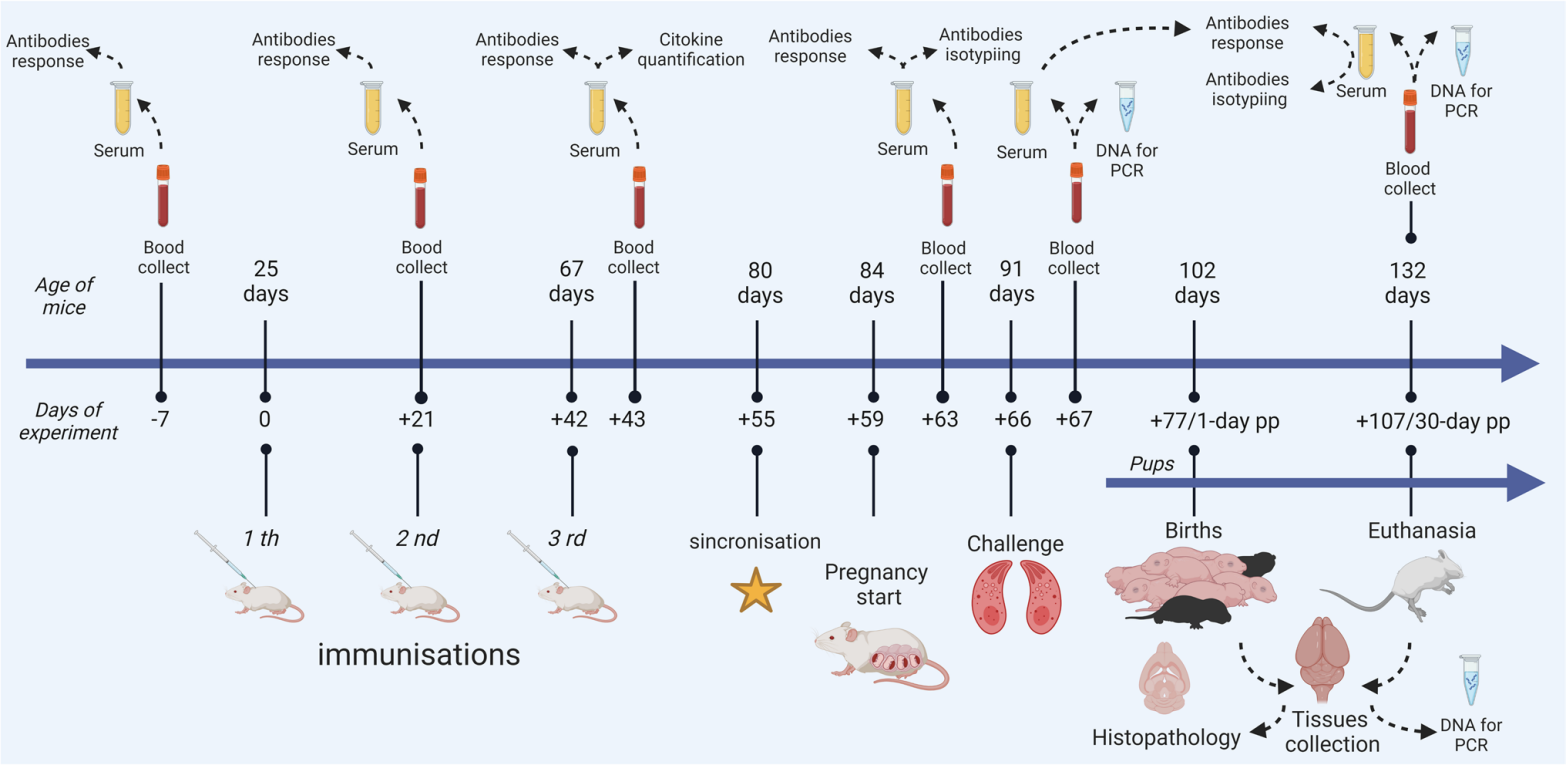
Timeline of the study showing the activities in each period (in days)

To evaluate different vaccine formulations using the recombinant peptides, 40 female BALB/c mice were used at 25 days of age, divided into eight groups of five animals. After birth, these BALB/c mice were housed in a maximum barrier isolator system in the Veterinary Department of the Universidade Federal de Viçosa (UFV, MG, Brazil). Groups of five females were individually restricted in ventilated cages (IVCs) with autoclaved wood shavings, placed in biosafety cabinets (Alesco^®^) at room temperature (20 °C), fed sterile chow and filtered water *ad libitum* under a 12-h light/dark photoperiod. These animals were immunised subcutaneously at three time points with an interval of 21 days after the first immunisation, using a volume of 200 μL per vaccine formulation, composed of peptides at different doses, a diluent (PBS pH 7.4) and the adjuvant saponin (Table 1, Fig. 1).

**Table 1:** Vaccine formulations for study groups

Groups		Vaccine formulation	Challenge
NC	Negative control	PBS	-
PC	Positive control	PBS	2 × 10^5^ tachyzoites
A	rsNcSAG4*	PBS + 50 μg saponin + 25 μg rsNcSAG4	2 × 10^5^ tachyzoites
B	rsNcGRA1*	PBS + 50 μg saponin + 25 μg rsNcGRA1	2 × 10^5^ tachyzoites
C	Combination*	PBS + 50 μg saponin +12.5 μg rsNcSAG4 + 12.5 μg rsNcGRA1	2 × 10^5^ tachyzoites
D	rsNcSAG4^+^	PBS + 50 μg saponin + 50 μg rsNcSAG4	2 × 10^5^ tachyzoites
E	rsNcGRA1^+^	PBS + 50 μg saponin + 50 μg rsNcGRA1	2 × 10^5^ tachyzoites
F	Combination^+^	PBS + 50μg saponin +25 μg rsNcSAG4 + 25μg rsNcGRA1	2 × 10^5^ tachyzoites

*Peptide used in low doses. ^+^Peptide used in a standard dose

### *N*. *caninum* tachyzoite preparation

The NC-1 tachyzoites were isolated from an infected dog and preserved in the Universidade de São Paulo Veterinary Department. The tachyzoites were propagated in African green monkey kidney cells (VERO-ATCC CCL-81) harvested with 80% monolayer confluence using sterile MEM AT081 medium (Himedia AT081) pH 7.2 and supplemented with 10% foetal bovine serum at 37 °C in 5% CO_2_ atmosphere. We confirmed the tachyzoite multiplication by observing the flask culture at 48-h intervals using inverted microscopy (Olympus IMT2). We cultured these tachyzoites from infected cells during continuous rounds of replications collected from those infected cells using cell scrapers and homogenised them using syringe passes. Cellular debris from this suspension of infected cells was centrifuged at 1350 × *g* for 15 min at 4 °C, resuspended the pellet in PBS pH 7.4 (NaCl 7.2 g, Na_2_HPO_4_ 1.48 g, NaH_2_PO_4_ 0.43 g, and MilliQ water 1000 mL) and centrifuged again to remove the cellular debris. We concentrated these tachyzoites by filtration using a syringe with a 5-μm membrane (Millipore^®^) and confirmed the viability of some aliquots in a passage in VERO cells using the abovementioned procedures. Some aliquots of concentrated tachyzoites were stored at −80 °C for use in PCR reactions as positive controls. We counted the number of viable tachyzoites by trypan blue exclusion, determined the mean by counting five samples of the final supernatant in a Neubauer chamber, and prepared the inoculum for challenged mice using 2 × 10^5^ tachyzoites in a volume of 100 μl.

### Challenge dams and vertical transmission assessment

Before the challenge, we synchronised the females for a suitable time of estrum 13 days after the third immunisation (day 55), keeping them in cages with bedding material from the cages of males 72 h before mating (Vandenbergh 1969). The presence of the vaginal mucoid plug determined the start of the gestational period after females matting for one night and 1 day (day 59). All pregnant dams except the negative control group were challenged subcutaneously with 2 × 10^5^ NC-1 tachyzoites on day 7 of gestation (day 66) according to the best gestation period of vertical transmission previously reported (López-Pérez et al. 2006; Jia et al. 2020). The productive infection in mice was confirmed using blood samples without EDTA collected 1 day after the protozoan inoculation (day 67), and we extracted the DNA and ran the PCR according to the method explained below. The birth generally started after 18 days of gestation (day 77), and we evaluated the number of animals per litter, newborn mortality, postnatal mortality, neurological signs, and other signs compatible with vertical transmission. The newborn mortality was calculated, including the pups dying 48 h after birth, and the postnatal mortality was calculated using the pups dying between 3 and 30 days post-partum (pp). The vertical transmission was confirmed by PCR using DNA extracted from tissue samples preserved at −80 °C collected from naturally dead and euthanised pups at day 30 pp (Fig. 1).

### *N*. *caninum* detection by PCR

PCR was conducted to evaluate the acute and chronic infection rates in dams and vertical transmission in pups. DNA from dams were obtained from blood samples collected 1 day after the challenge and at the end of the experiment, and the tissue samples were collected at the end. DNA from pups were extracted from dead and surviving euthanised mice at 30 days pp (Fig. 1). The genomic DNA was extracted from tissues and blood samples using the Wizard kit (Promega^®^) following the manufacturer’s instructions. We performed the PCR process according to the method previously standardised by Blanco Martinez (2013) with some modifications. Briefly, we used the primers involved in the coding region of NcMIC4 (GenBank: BK005222) that expressed during tachyzoite host cell invasion, NcMIC4-F (5′AGGTCAAAGAGCTGCGATA 3′) and NcMIC4-R (3′AGGAACCCCTTTCCTCTGAA 5′), generating an amplicon of 198 pb. In each reaction, the mix included 1 μl of genomic DNA, 12.5 μl of Go Taq Green master mix (Promega), 1 μl of primers F and R, and 10.5 μl of H_2_O-free. We used DNA from the culture of tachyzoites and DNA from non-infected Vero cells as positive and negative controls. The thermocycling protocol was 94 °C for 1 min, 35 cycles of 94 °C for 30 s, 48 °C for 35 s, and 72 °C for 5 min.

### ELISA

Blood samples from each mouse were collected by orbital plexus puncture on day 7 before the first immunisation and on days 21, 43, 63, 67, and 107 after starting the immunisation regime (Fig. 1), separating the serum by centrifugation at 3000 × *g* for 15 min at 4 °C. Briefly, we performed ELISA coating 0.5 μg/well of rsNcGRA1 and rsNcSAG4 in separate plates Maxisorp Nunc^®^. We used the solution of 2% casein diluted in PBS to block the non-specific sites of ligation, adding the serum samples in the dilution 1:200 in an incubation buffer with 2% casein and Tween 20 in triplicate. We used a secondary rabbit anti-mouse IgG antibodies conjugated with horseradish peroxidase (Sigma^®^, diluted 1:8000 in incubation buffer) to recognise the serum antibodies. We revealed the plates using OPD (o-phenylenediamine dihydrochloride) as a substrate, measuring the absorbance using an ELISA reader (Thermo Scientific) at 492 nm. We considered a sample positive when the optical density (OD) was higher than the mean value plus three standard deviations of the OD obtained for negative controls.

We identified the antibody isotypes qualitatively using the serum from blood samples collected on days 63 and 107 after the immunisation program (Fig 1). We used the goat monoclonal antibodies against mice IgG1, IgG2a, IgG2b, IgG3, IgM, and IgA from the ISO-2 kit (Sigma^®^) in the reactions using Maxisorp Nunc^®^ plates, adding the IgGs from serum samples. Rabbit anti-goat IgG conjugated with horseradish peroxidase (Sigma^®^) was used as a secondary antibody to reveal the ELISA reactions. Following the manufacturer’s instructions, we confirmed each reaction's positivity by visualising the plate.

### BD cytometric bead array (CBA) analysis

We quantified the cytokine production using the serum samples collected 1 day after the third immunisation (day 43) from each dam of all groups (Fig. 1). We processed these samples by mice Th1/Th2/Th17 cytokine kit (No. 560485, BD Biosciences) according to the manufacturer’s instructions. We mixed 50 μL of each serum sample with 50 μL of capture beads and loaded it into a microtube, incubating it in the dark for 2 h. After adding 300 μL of water, we tested the serum cytokine levels of Th1 (IL-2, TNF, and IFN-γ), Th2 (IL-4, IL-6, and IL-10), and Th17 (IL-17A) using a BD FACSCanto™ flow cytometer and finally analysed the data using BD Cytometric Bead Array analysis software.

### Histopathology

Histopathology was carried out using the tissues collected from euthanised females at the end of the experiment (day 107) (Fig. 1); some offspring pups died, and others were euthanised. We performed the euthanasia in mice using sodium pentobarbital at 100 mg/kg, collecting the tissue samples after animal dissection and conserving tissues for PCR at −80 °C. We fixed the tissues in paraformaldehyde 4% solution (pH 7.4) for 24 h, and then they were dehydrated in an increased graded series of ethanol concentrations, diaphanised in xylene, and embedded in Paraplast (Sigma^®^). We sectioned these tissues at 4 μm of thickness, mounted them on glass slides, and stained them with haematoxylin-eosin (H&E), and the slides were examined using an optical microscope (Nikon ECLIPSE E6003). The brain injuries were assessed by analysing two sagittal sections of each brain and classified according to an injury scoring system of (0) without injuries, (1) mild (small inflammatory foci, perivascular cuff, absence of necrosis), (2) moderate (more significant inflammation and foci with necrosis), and (3) severe (foci of necrosis greater than 5% of the parenchyma) (Rettigner et al. 2004).

### Statistical analysis

We calculated the differences between groups in the results of the ELISA assays using a nested one-way ANOVA test, using the mean OD values between serum samples from different groups. We analysed the kinetics of antibodies over time using a two-way ANOVA test, using the mean OD values from samples collected on different days. We used the Tukey multiple comparison test to verify the differences detected by the ANOVA tests, and the results with *p* < 0.05 were considered statistically significant. We calculated the differences in cytokine response between groups using the Kruskal-Wallis test at *p* < 0.05, and differences in cytokine response between the groups were analysed using Dunn’s multiple comparisons test at *p* < 0.05. We used the Kaplan–Meier method to estimate the percentage of mice surviving at each time point after the challenge, comparing the survival curves by log-rank test. We carried out the statistical analyses using GraphPad Prism 8.0.1^®^ software.

## Results

### Yeast transfection and recombinant peptide confirmation

**Fig. 2:**
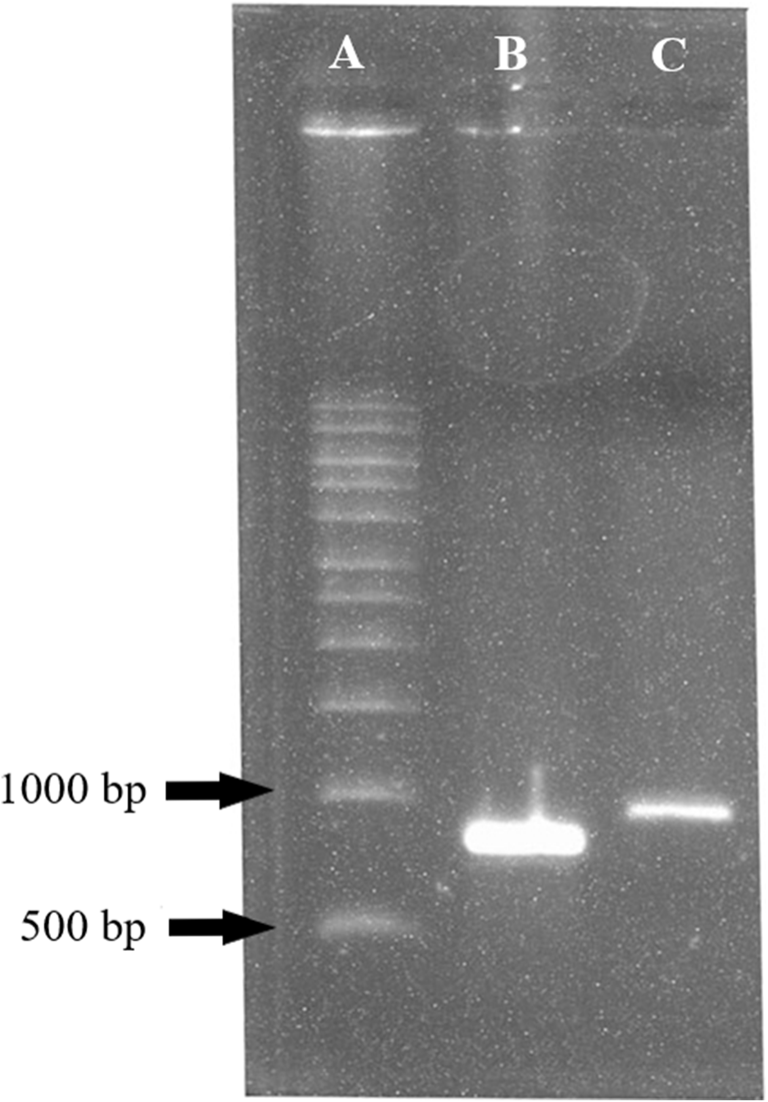
Agarose gel by PCR product showing the stable insertion of the expression cassette by colony PCR of pre-selected MD growing clones using the AOX1-promoter and terminator primers (5′-AOX1-f and 3′-AOX-r). (A) Molecular weight marker. (B) The PCR product of *K. phaffii* transformed with rsNcGRA1 showing the band near 919 bp (269 bp of minigene coding to rsNcGRA1 + 650 bp of pPIC9k vector). (C) The PCR product of *K. phaffii* transformed with rsNcSAG4 showing the band near 973 bp (323 bp of minigene coding to rsNcSAG4 + 650 bp of pPIC9k vector)

The transformed yeast His^+^ selected clones that grew for 2–3 days on selective medium MD after inserting the transfection cassette showed that the histidinol dehydrogenase gene, involved in histidine synthesis, was only present in the transfected yeast. The resistant geneticin clones that grew at the higher geneticin concentration (1 g/mL) showed multiple copies of the exogenous genes inside the Km71 yeast genome. The colony PCR showed the amplification of DNA fragments composed of 919 bp (rsNcSAG4) and 973 bp (rsNcGRA1), confirming the adequate insertion of the transformation cassette into the genome of transformed clones **(**Fig. 2**)**. The transformed clones with the AOX1 gene showed an adequate multiplication during the fermentative process described in the “Fermentative process of recombinant peptide production” section and the induction of peptide expression by methanol addition throughout 96 h until the end of the fermentative process, in association with this inserted gene.

**Fig. 3:**
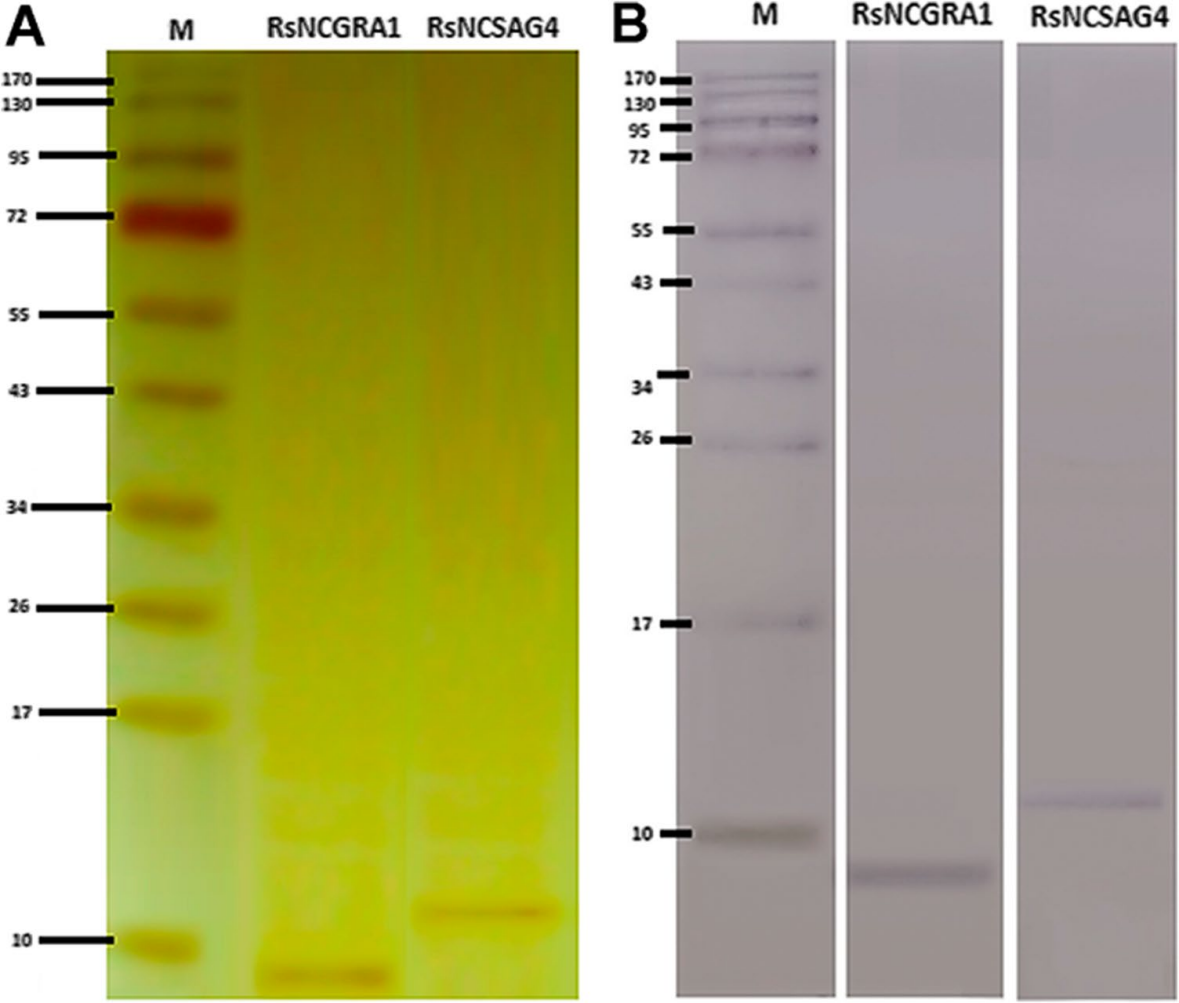
Specific bands of rsNcSAG4 (10.5 kDa) and rsNcGRA1 (8.9 kDa) after tangential flow filtration. **A** Silver-stained SDS-PAGE electrophoresis in 16% of concentration **B** in Western Blotting using rabbit polyclonal IgG anti-*N.caninum* diluted in 1:200. M, molecular weight marker

In the transformed yeasts genome, the adequate incorporation of a signal peptide responsible for exporting each recombinant peptide to the culture medium allowed this recovery by centrifugation of supernatant of the batch fermentations, facilitating the purification and sterilisation by tangential flow filtration. The recombinant peptides obtained from the filtered supernatant were analysed by SDS-PAGE and western blotting, confirming the specific bands around 11 kDa and 9 kDa of rsNcSAG4 and rsNcGRA1, respectively. In the western blotting, these peptides showed recognition by rabbit anti-*N. caninum* polyclonal antibodies diluted 1:200 **(**Fig. 3**)**. The samples of filtered supernatant showed no growth after submitting them to Sabureau medium and blood agar, confirming the microbial safety of the solution with recombinant peptides. Finally, the quantification of the final product by bicinchoninic acid assay (BCA) resulted in 3.2 μg/μL of rsNcSAG4 and 4.6 μg/μL of rsNcGRA1 for vaccine formulations.

### Antibody response

#### Antibodies response against rsNcSAG4 antigen

**Fig. 4:**
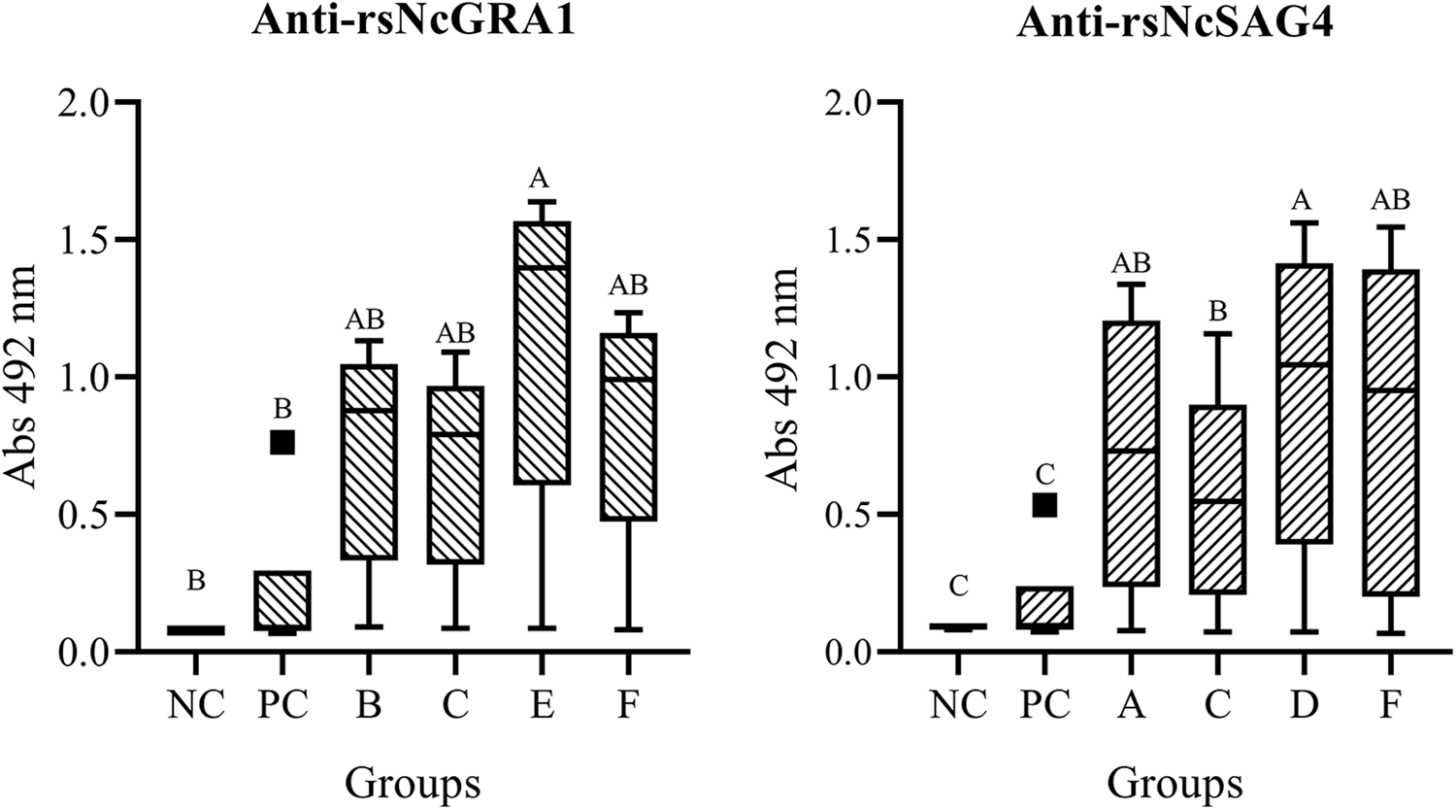
Comparison of ELISA tests showing the boxes (mean values), whiskers, and extreme values analysed by nested one-way ANOVA showing the Tukey multiple comparison difference at *p* < 0.05 of significance by different letters

In the serum samples of dams that received this peptide in the vaccine formulations at a low or standard dose, the nested one-way ANOVA test and Tukey test with the mean OD values of sera from animals immunised with rsNcSAG4 at a low dose (25 μg, group A), at a standard dose (50 μg, group D), in combination with rsNcGRA1 at a low dose (12.5 μg, group C), and in combination with rsNcGRA1 at a standard dose (25 μg, group F) showed a statistically significant difference (*p* < 0.05) in comparison to the values of the negative and positive controls. The comparison between immunisation groups A, D, F showed no statistically significant difference (*p* ˃ 0.05). However, the mean OD values of sera from animals immunised with rsNcSAG4 at a standard dose (50 μg, group D) showed a statistically significant difference (*p* < 0.05) in comparison to the values of animals immunised with the same peptide in combination at a low dose (12.5 μg, group C) **(**Fig. 4**)**.

#### Antibodies response against rsNcGRA1 antigen

In the serum samples of dams that received this peptide in the formulation at a low or standard dose, the nested one-way ANOVA test and Tukey test using the mean OD values of sera from animals immunised with rsNcGRA1 at a low dose (25 μg, group B), at a standard dose (50 μg, group E), in combination with rsNcSAG4 at a low dose (12.5 μg, group C), and standard dose (50 μg, group F) showing a statistically significant difference (*p* < 0.05) in comparison to the values of negative and positive controls. The mean OD values of animals immunised with rsNcGRA1 at a standard dose (50 μg, group E) showed a statistically significant difference (*p* < 0.05) in comparison with mean OD values of animals immunised with this peptide at a low dose (25 μg, group B), combination at a low dose (group C) and combination at a standard dose (group F). However, the mean OD values of animals immunised with rsNcGRA1 at a low dose (group B) were not significantly different in comparison with values of animals immunised with this peptide in combination with a low dose (group C) and combined at a standard dose (group F). The mean OD values of animals immunised with rsNcGRA1 in combination at a low dose (group C) and combination at a standard dose (group F) did not show a statistically significant difference (*p* ˃ 0.05) **(**Fig. 4**)**.

#### Kinetics antibodies

The groups immunised with the peptides rsNcGRA1 and rsNcSAG4 showed the same kinetic pattern on days. The mice immunised with these peptides at a low dose and standard dose by ELISA tests generated serum OD mean values that increased gradually according to the collection day, with a significant difference (*p* < 0.05), with the highest values in samples from animals immunised with peptides at a standard dose. Twenty-one days after the first immunisation, the levels of antibodies significantly increased (*p* < 0.05) compared to the mean OD values of serum samples collected on day 7. Similarly, 21 days after the second immunisation (day 43), the levels of antibodies significantly increased (*p* < 0.05) compared to the mean OD values of serum samples collected on day 21. The highest IgG peak was reached 21 days after the third immunisation (day 63), and these OD values were significantly higher (*p* < 0.05) than both values at 21 days after the second (day 43) and first immunisations (day 21).

**Fig. 5:**
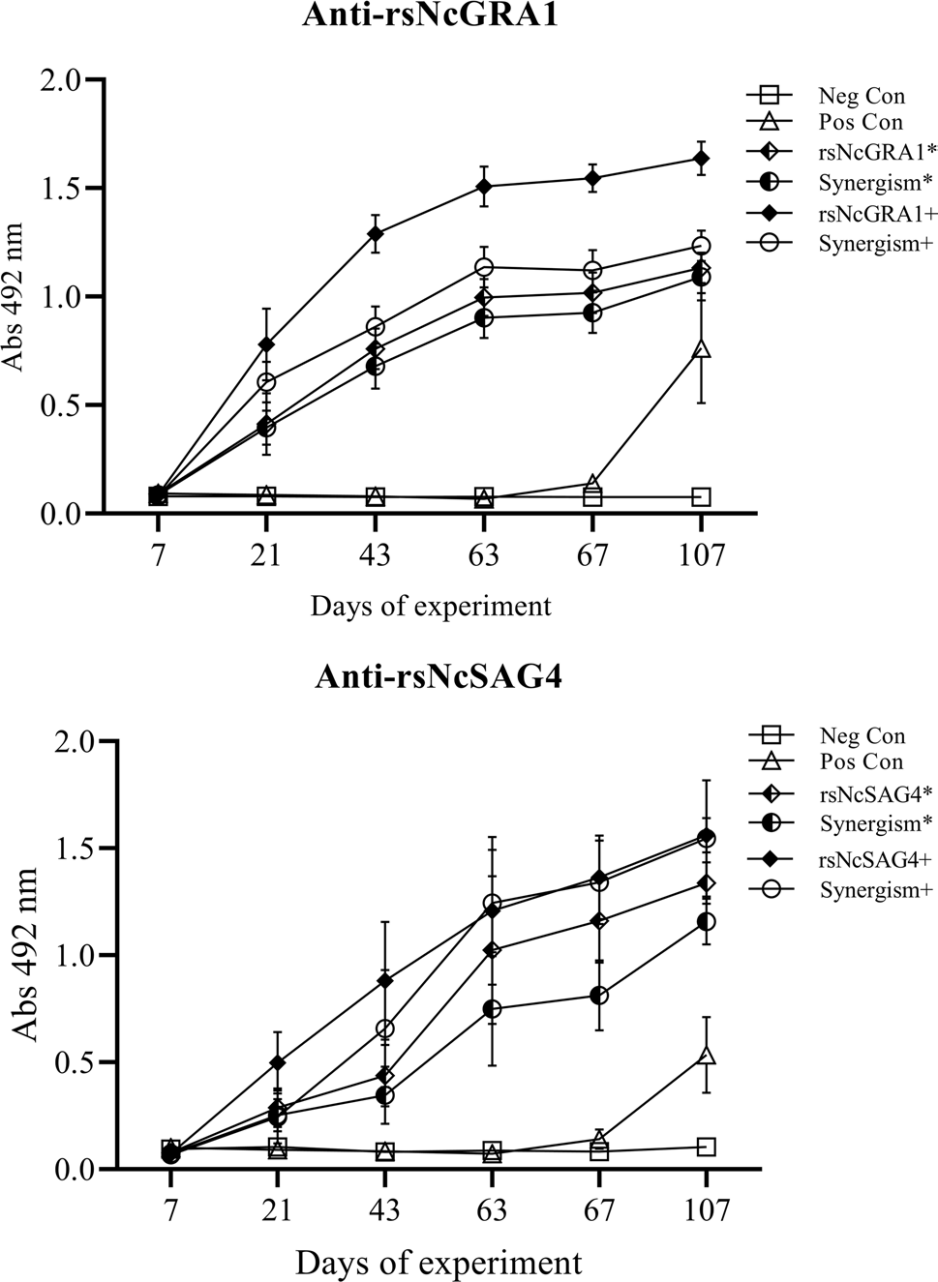
Kinetics of antibodies in serum samples from mice of each study group (sera diluted 1:200) showing the mean of optical density (OD) and standard deviation after vaccination at three time points with an interval of 21 days

The samples collected from negative control mice were not statistically significantly different (*p* ˃ 0.05) on different collection days. However, the samples from positive control animals were significantly different at day 107 compared to day 67, with the mean OD values increasing gradually from 7 days after the challenge (day 67) and reaching their highest value 47 days after the challenge (day 107). The samples collected on previous days showed no significant difference, which remained near 0. In parallel, the antibody levels were significantly increased (*p* < 0.05) at day 67 after the challenge in dams immunised with NcGRA1 at a low dose and dams immunised with peptides in combination at a low dose. However, the dams immunised at a standard dose for the other immunisation groups did not show a significant difference after the challenge (*p* > 0.05) between the samples collected on day 107 and day 67 **(**Fig. 5**)**.

#### Antibody isotyping

The antibodies of animals immunised with peptides at a low dose and standard dose collected 21 days after the third immunisation (day 63) showed a humoral response, with a reaction to IgG1. The control groups did not show a reaction to IgG1 at this time. At the end of the experiment, only the serum of mice immunised with peptides at a standard dose, collected 63 days after the third immunisation (day 107), showed the same humoral response, with a reaction to IgG1. At the same time, the antibodies of animals immunised with peptides at low doses and positive controls showed a reaction to both IgG1 and IgM. However, at the end of the experiment, the negative control mice sera did not react to any antibody isotype. However, the samples analysed did not detect the isotopes IgG2a, IgG2b, IgG3, and IgA.

### Cytokine response

**Fig. 6:**
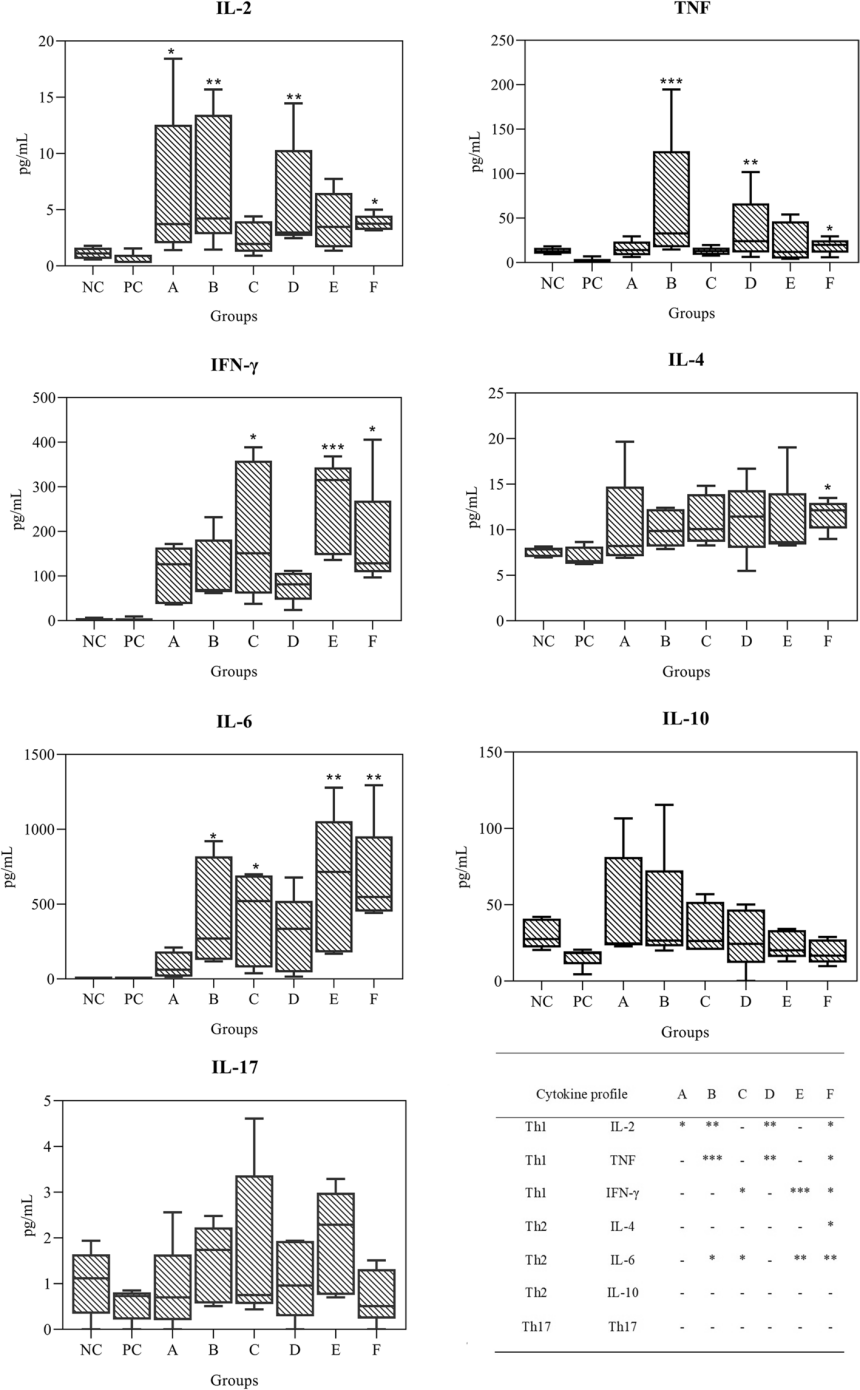
Serum cytokine profile in non-pregnant mice (43 dpi) of all study groups. By Dunn’s multiple comparisons tests, boxes represent the median, whiskers, and individual points represent extreme values. (*) and (**) indicate Kruskal-Wallis, *p* < 0.05, and statistically significant differences compared to controls

The serum levels of Th1 (IL-2, TNF, and IFN-γ), Th2 (IL-4, IL-6, and IL-10), and Th17 (IL-17A) cytokines were measured using a BD FACSCanto™ flow cytometer on day 43, 36 h after the third immunisation, before the challenge. Concerning the serum Th1 cytokine profile, the IL-2 levels in mice immunised with peptides rsNcSAG4 and rsNcGRA1 formulated at a low dose and standard dose (groups A, B, D, and F) were significantly different from the positive control (*p* < 0.05), with a highly significant difference in mice immunised with rsNcGRA1 and rsNcSAG4 (groups B and D) at a low dose and standard dose, respectively. The serum TNF was significantly different from the positive control (*p* < 0.05) in mice immunised with rsNcGRA1 at a low dose, rsNcSAG4 and combination at the standard dose (groups B, D, and F), with a highly significant difference in mice immunised with rsNcGRA1 at low dose. The IFN-γ was significantly different (*p* < 0.05) to both controls in mice immunised with peptides together at low and standard doses and rsNcGRA1 at the standard dose (groups C, E, and F), with a highly significant difference in mice immunised with peptides in combination at a standard dose (group E). In the evaluation of the serum Th2 cytokine profile, the IL-4 only showed a significant difference (*p* < 0.05) in mice immunised with peptides in combination at a standard dose (group F) in comparison to the positive control. In contrast, the IL-6 level showed a significant difference (*p* < 0.05) in mice immunised with peptide rsNcGRA1 and combination at a low and standard dose (groups B, C, E, and F) in comparison to both controls, with a highly significant difference in mice immunised with rsNcGRA1 and combination at a standard dose (group E and F). IL-10 and serum IL-17 levels did not significantly differ (*p* ˃ 0.05) between the immunisation and control groups. A balanced Th1/Th2 cytokine profile with statistically significant production of both Th1 and Th2 cytokine was identified in mice immunised with peptides in combination at a standard dose (group F) and in mice immunised with peptide rsNcGRA1 at a standard dose and peptides in combination at a low dose (groups E and C). The Th1 cytokine profile was most dominant in mice immunised with rsNcSAG4 at a standard dose (group D) and in mice immunised with peptides rsNcSAG4 and rsNcGRA1 at a standard dose (groups B and A) **(**Fig. 6**)**.

### Assessment of the *N*. *caninum* multiplication in pregnant dams

**Fig. 7:**
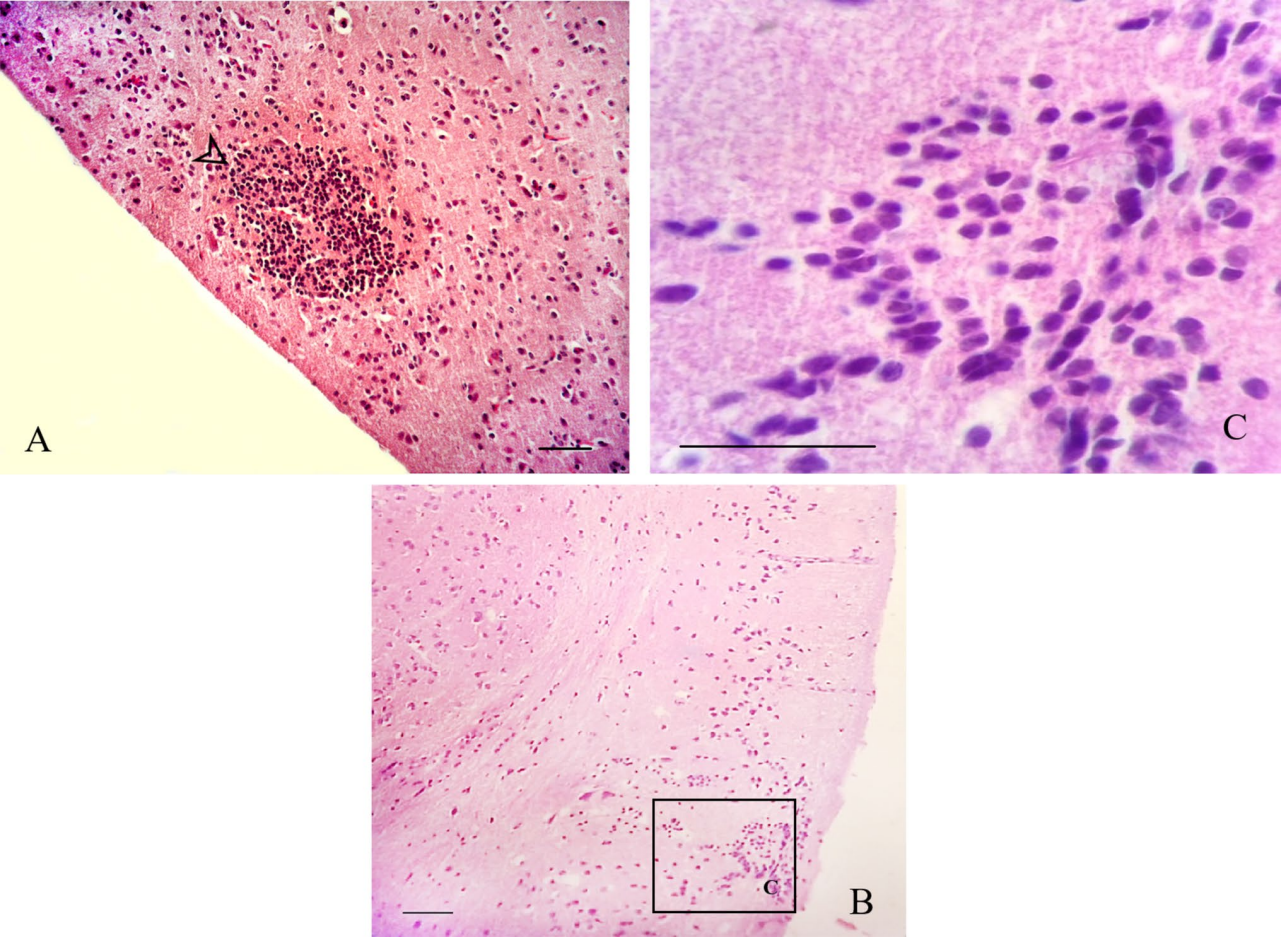
Cerebral histopathology, scale bar 100 μm. Microphotography by H&E stain showing **A** small necrotic foci surrounded by cells in gliosis in the brain of the dam (arrow), pup injured area with cells in gliosis and **B** gliosis foci with **C** more magnification

All pregnant dams in the experiment exhibited acute parasite multiplication. However, in the pregnant-challenged dams of positive control, the physical evaluation did not identify any dead mice or neurological signs; these dams showed apathy and hirsute hair with 80% morbidity. These dams showed 80% chronic infection with a 20% reduction in parasite detection between acute and chronic (Table 2). In these dams, the histopathology of the brain tissues revealed mild lesions in four animals with small foci of gliosis. Only one dam showed a moderate cerebral lesion score, with focal necrosis and multifocal gliosis **(**Fig. 7A**)**. Also, this animal showed mononuclear cell foci in the myocardium and renal cortex and degenerative-necrotic changes in the liver.

**Table 2:** Health parameters observed in challenged dams of experimental groups

Group	Pregnancy rate^a^	Little size^b^	Morbidity rate^c^	Mortality rate^d^	Cerebral lesions^e^	Acute parasite detection (D + 1)^f^	Chronic parasite detection (D + 107)^g^	Reduction of parasite detection
Negative control	5/5 (100%)	12.4 ± 1.7	0/ 5(0%)	0/5 (0%)	0	0/5 (0%)	0/5 (0%)	
Positive control	5/5 (100%)	10. 8± 2.4	4/5 (80%)	0/5 (0%)	1.2	5/5 (100%)	4/5 (80%)	20%
rsNcSAG4*	4/5 (80%)	11.0 ± 3.8	2/4 (50%)	0/4 (0%)	0	4/4 (100%)	2/4 (50%)	50%
rsNcGRA1*	2/5 (40%)	5.0 ± 4.2	2/2 (100%)	0/2 (0%)	0.2	2/2 (100%)	2/2 (100%)	0%
Combination*	4/5 (80%)	9.8 ± 2.5	2/4 (50%)	0/4 (0%)	0	4/4 (100%)	2/4 (50%)	50%
rsNcSAG4^+^	3/5 (60%)	10.7 ± 0.6	0/3(0%)	0/3 (0%)	0	3/3 (100%)	1/3 (33%)	67%
rsNcGRA1^+^	4/5 (80%)	10.5 ± 3.0	0/4 (0%)	0/4 (0%)	0	4/4 (100%)	1/4 (25%)	75%
Combination^+^	5/5 (100%)	8.2 ± 1.1	0/5 (0%)	0/5 (0%)	0	5/5 (100%)	0/5 (0%)	100%

^a^No of pregnant mice/mice housed with males (percentage)

^b^The average number of full-term delivered pups (±SD)

^c^No dams showing clinical signs/no dams in the group (percentage)

^d^No of dead dams before day 30 pp /No dams in the group (percentage)

^e^The average value of cerebral score lesions

^f^No of blood PCR positive dams/no dams in the group (percentage)

^g^No of brain PCR positive dams/no dams in the group (percentage)

The dams immunised with peptides at a low dose showed mild neurological signs with apathy 5 days after the experiment, exhibiting morbidity ranging between 50 and 100%, just like the positive controls. These dams showed chronic infection, with a positivity rate ranging between 50 and 100%, and a cutback of parasite detection from 0 to 50 % in the acute and chronic stages. These dams revealed a depletion in pregnancy with a low fertility rate (40%) in dams immunised with rsNcGRA1 associated with a high chronic infection rate (100%) **(**Table 2**)**. One of these groups showed mild cerebral injury with a low brain score and small gliosis foci without injury in the other tissues analysed.

The dams immunised with peptides at a standard dose did not identify any clinical or neurological signs with 0% morbidity. The dams immunised with rsNcSAG4 and rsNcGRA1(groups D and E) showed a low chronic infection rate of 33 and 25%, and these dams had a reduction of the parasitic rate of 67 and 75%, respectively. Also, the dams immunised with rsNcSAG4 showed a moderate fertility rate (60%). However, the dams immunised with combined peptides showed a 100% reduction in parasite detection without developing a chronic infection **(**Table 2). The histopathology in these animals revealed a normal brain, myocardium, kidney, and liver structure.

### Assessment of the infection of *N*. *caninum* in pups

The pups of the positive control dams showed apathy and hirsute hairs without neurological signs in the clinical evaluation. These pups showed early-born mortality of 13% and postnatal mortality of 21%, with overall mortality per pup of 31%. The vertical transmission in dead and those that survived reached 81% (Table 3). Histopathological assessment of the brains of these pups revealed mild lesions with small foci of gliosis without structures compatible with free tachyzoites and tissue cyst formations (Fig. 7B–C).

**Table 3:** Health parameters observed in pup’s offspring dams of experimental groups

Group	Clinical signs^a^	Early-born mortality^b^		Post-natal mortality^c^		Overall mortality	Cerebral lesions^d^	The infection rate in dead pups^e^	Infection rate in surviving pups^f^	Vertical transmission rate
		Per pup	Per litter	Per pup	Per litter	Per pup				
Negative control	-	0/62 (0%)	0/5 (0%)	0/62 (0%)	0/5 (0%)	0/62 (0%)	0	0	-	
Positive control	+ (apathy and hirsute hair)	7/54 (13%)	2/5 (40%)	10/47 (21%)	3/5 (60%)	17/54 (31%)	1	12/17 (70%)	26/37 (70%)	38/47 (81%) ^g^
rsNcSAG4*	+ (apathy and hirsute hair)	0/44(0%)	0/4 (0%)	2/44 (5%)	2/4 (50%)	2/44 (5%)	0	1/2 (50%)	-	50%
rsNcGRA1*	+ (apathy and hirsute hair)	0/10 (0%)	0/2 (0%)	0/10 (0%)	0/2 (0%)	0/10 (0%)	0	0	10/10 (100%)	100%
Combination*	+ (hirsute hair)	2/39 (5%)	1/4 (25%)	0/37 (0%)	0/4 (0%)	2/39 (5%)	0	2/2 (100%)	-	100%
rsNcSAG4^+^	-	0/32 (0%)	0/3 (0%)	0/32 (0%)	0/3 (0%)	0/32 (0%)	0	0	2/32 (6%)	6%
rsNcGRA1^+^	-	0/42 (0%)	0/4 (0%)	0/42 (0%)	0/4 (0%)	0/42 (0%)	0	0	0/42 (0%)	0%
Combination^+^	-	0/41 (0%)	0/5 (0%)	0/41 (0%)	0/5 (0%)	0/41 (0%)	0	0	0/41 (0%)	0%

^a^Main clinical sign observed in pups

^b^Number of stillborn and dead pups until day 2 pp/total delivered pups (percentage)

^c^Number of dead pups from day 3 pp onwards/ No of pups alive by day 2 pp (% pup mortality)

^d^Average value of cerebral score lesions

^e^PCR-positive of dead pups/no of dead pups (percentage)

^f^PCR-positive of surviving pups/no of surviving pups at 30-day pp (percentage)

^g^PCR-positive of dead and surviving pups/no of surviving pups at 3-day pp (percentage)

Concerning the pups of dams immunised with peptides at a low dose, the pups from those immunised with rsNcSAG4 (group A) showed an overall mortality of 5%. Similarly, the offspring from dams immunised with peptides in combination (group C) showed a mortality rate of 5%. The clinical evaluation of these animals demonstrated apathy and hirsute hairs without neurological signs. The vertical transmission revealed high vertical transmission rates of 50% in the offspring from dams immunised with rsNcSAG4 (group A), 100% in pups from dams immunised with peptide rsNcGRA1 (group B), and 100% in pups from dams immunised with peptides in combination (group C) (Table 3). The offspring of dams immunised with peptide rsNcGRA1 (group B) showed a small, but statistically significant (*p* ˂ 0.05) mortality reduction compared to other groups immunised with peptides at low doses (Table 4).

**Table 4:** Survival parameters in pups from experimental groups

Experimental group	Pup survival rate	*p*-value of survival
Negative control	100%	<0.001*
Positive control	69%	Control
rsNcSAG4*	95%	<0.001*
rsNcGRA1*	100%	0.0506
Combination*	95%	<0.001*
rsNcSAG4^+^	100%	<0.001*
rsNcGRA1^+^	100%	<0.001*
Combination^+^	100%	<0.001*

The survival curve in pups was analysed using the Kaplan-Meier test and compared with the positive control (log-rank test). The group with a *p* < 0.01 (*) was considered statistically significant

**Fig. 8:**
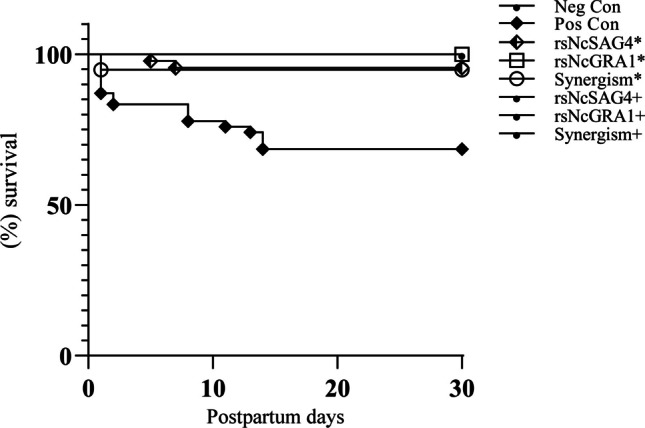
Pup survival curve of study groups after 30 days of birth using the Kaplan–Meier method

Concerning the pups of dams immunised with peptides at standard doses, these animals survived throughout the experimental period without mortality, neurological or clinical signs. Similarly, the surviving pups from dams immunised with the standard dose of peptide rsNcGRA1 and, in combination, did not show vertical transmission. However, only the pups from dams immunised with rsNcSAG4 showed a low vertical transmission rate (6%) (Table 3). These pups showed statistically significant (*p* ˂ 0.05) mortality reduction concerning positive control (Table 4). Finally, the offspring survival analysis in all immunised groups showed a significant increase in the time of survival (*p* < 0.05) compared to the positive control animals (Fig. 8).

## Discussion

This study demonstrated for the first time that peptides predicted by T and B epitopes of native antigens of *N. caninum* could be efficiently expressed in recombinant *K. phaffii*, inducing an appropriate immune response in dams reducing the chronic infection and vertical transmission in pups of BALB/c mice. During the recombinant process, the transfection cassette showed crossing-over substituting the yeast genome’s homologous regions, confirmed by colony PCR of the transformant yeast (Cereghino et al. 2001). As shown in this study, the expression cassettes containing the AOX1 promoter transformed the yeast by a single crossover event substituting AOX1 or His4 loci insertion into the yeast genome, inducing a His^+^ expression phenotype (Cregg et al. 2000). The AOX1 gene inserted in yeast allowed the methanolic induction of heterologous genes because the Km71 strain has a partially-deleted AOX1 gene replaced by the Arg4 gene of *Saccharomyces cerevisiae* (Cereghino and Cregg 2000)*.* The α-mating factor (MATα) secretion signal in the transfection cassette under the control of the AOX1 promoter exported the recombinant peptides to the extracellular yeast culture medium after induction with methanol (Joo et al. 2017).

The recombinant peptides have been easily obtained from the extracellular medium due to the low secretion of yeast native proteins ranging between 21.82 and 109.45 kDa, in contrast to recombinant peptides at around 10 kDa (Cregg et al. 1993; Mattanovich et al. 2009). Consequently, transfected yeast secreted high titres of relatively pure heterologous peptides after fermentation (Cregg et al. 2000) because the AOX1 promoter improves the folding, leading to very high expression levels and tight regulation of recombinant peptides (Vogl et al. 2013). The culture medium centrifugated and filtered by cartridge membranes, including membranes of 0.45 and 0.22 μm, was negative for microorganism growth, agreeing with the complete sterilisation of solutions using filters of 0.2 μm (van Reis et al. 1991). After this process, the peptides were carefully separated and confirmed by SDS-PAGE and Western blotting, verifying their expression, accurate folding, and antigenicity.

Those recombinant peptides administered individually or in combination, at low and standard dosages, elicited a humoral immune response; however, the peptides at a standard dose showed the highest antibody titres in animals immunised with them. Among these, the rsNcGRA1 peptide in the individual formulation at standard doses showed the highest titre of antibodies among all the immunisation groups. This response could be related to predicted O-glycosylation sites, with five sites for rsNcGRA1 and three sites for rsNcSAG4 (Silva de Araujo 2017) that could result as a consequence of post-translational modification in the *K. phaffii* heterologous expression system (Macauley-Patrick et al. 2005). This plausible property favours the immunogenic features of rsNcGRA1 compared to rsNcSAG4 because glycosylation contributes to antigen recognition and processing. In this scenario, the APCs become the primary source of IL-6, playing an essential function in T cell differentiation related to the highest levels of IL-6 observed in dams immunised with standard doses of rsNcGRA1, both individually and in combination (Rincón et al. 1997). T cell differentiation is related to the high level of IL-4 produced by those dams immunised because this is generally produced by differentiated and amplified CD4^+^ T cells stimulated by IL-6 after priming undifferentiated Th 0 cells (Rincón et al. 1997).

The dams immunised with standard doses of rsNcGRA1 alone and in combination reacted to IgG1, with a Th2 profile after the third immunisation inducing significant levels of IL-6 and IL-4. The IL-6 both promoted the differentiation of T-cells and B-cells to plasmablasts leading to the secretion of high levels of IgG1 due to Th 0 differentiation (Kishimoto 1989; Lue et al. 1991). In the same dams, the expression of IL-4 after the third immunisation suggests the differentiation of naive CD4^+^ T cells into IL-4-producing effector Th2 cells by IL-6 induction (Rincón et al. 1997). The increased antibody titres in dams immunised with standard doses of rsNcGRA1 indicate that IL-4 was produced after gradual differentiation of CD4^+ ^T for Th2 cells. Hence, the production of IL-4 is essential for the induction of protective immunity against *N. caninum,* related to clonal amplification and somatic hypermutation of T and B cells.

The persistence of high titres of antibodies observed after the challenge with the protozoan infers the induction of memory B-cells after immunisation with these peptides at a standard dose because the B-cells could respond quickly, secreting IgG1 after the challenge (Bernasconi 2002). These facts relate to the best health status without cerebral parasite multiplication observed in pups from dams immunised with standard doses of rsNcGRA1, individually and in combination. In those dams, the highest antibody titters with a reaction of IgG1 observed after the challenge contribute to progressive parasite protection. In this sense, the IgG1 contributes to tachyzoite opsonisation for parasite neutralisation at the early stage of infection (Nishikawa et al. 2001a) and participates in controlling vertical transmission, leading to the highest rate of pup survival and low mortality rate observed in standard dose-immunised dams (Nishikawa 2017).

Since the IgG1 alone cannot protect the dams and pups from *N. caninum* progression, the CD4^+^ and CD8^+^ T cells must be essential in preventing *N. caninum* intracellular multiplication at the early and chronic stages of infection (Nishikawa et al. 2001a; Nishikawa 2017). On the other hand, in dams immunised with standard doses of rsNcGRA1, individually and in combination, the balance between Th1 and Th2 showed the production of Th1 profile cytokines, with the increase of IFN-γ and IL-2 levels, respectively, which showed an essential effector of immunity against intracellular multiplication with *N. caninum*. In this scenario, Th2 produced IL-6 in the presence of IL-2 and IFN-γ, suggesting the induction of CD8^+^ T cells and a Th1 immune response profile (Takai et al. 1988). The production of IFN-γ suggests differentiating CD8^+^ T cells into cytotoxic T cells (CTL) and IFN-γ production by CTL and CD4^+^ T cells after antigen stimulation (Puliaev et al. 2004). The plausible presence of CTL cells is associated with parasite reduction after the challenge in dams immunised with standard doses of rsNcGRA1, individually and in combination (Nishikawa 2017). The levels of IFN-γ are related to parasite destruction and pup survival parameters observed in these dams because macrophages activated by this exhibit killing activity against *N. caninum* associated with increased production of nitric oxide (NO). Previous reports show that mice deficient in the IFN-γ gene have been susceptible to *N. caninum* infection (Nishikawa et al. 2001b).

The low levels of anti-inflammatory IL-10 and IL-17 in groups immunised with standard doses suggest a low interference in the Th1/Th2 immune response balance identified in dams that received the peptides in combination or rsNcGRA1 alone. As a result, the appropriate balance between the Th1 and Th2 responses appears to be crucial in controlling parasite multiplication and maintaining a pregnancy (Nishikawa et al. 2003). The mixed profile of the immune response against *N. caninum* during pregnancy is a significant challenge for the development of vaccines because excessive stimulation of the Th1 response can be harmful and cause foetal death (Rosbottom et al. 2008). Also, an increase in parasite spread is associated with the lack of antibodies (Rojo-Montejo et al. 2011), and high expression of IFN-γ in vaccinated mice was associated with a lack of protection against challenge (Aguado-Martínez et al. 2009b). However, the balance of the Th1/Th2 immune responses observed in dams immunised with standard doses in combination or rsNcGRA1 is related to the health status of those dams and pups, with the absence of signs and lesions compatible with *N. caninum* infection. This clinical manifestation is related to controlling the vertical transmission, maintaining survival rates, and fertility in the dams immunised with rsNcGRA1 and peptides in combination at a standard dose. However, the dams immunised with rsNcGRA1 at a low dose and rsNcSAG4 at a standard dose showed the lowest rate of pregnancy associated with increasing levels of TNF-α, suggesting a plausible alteration in the gestation establishment by compromising the reproductive tract (López-Pérez et al. 2010; Tang et al. 2023). However, the dams immunised with peptides in combination at standard dose showed an immune response profile compatible with gestation establishment, revealing the best pregnancy rate.

However, concerning dosage, the peptides at a low dose elicited both low antibody titres and high-affinity antibodies. After the challenge, the peptides administered individually and in combination showed a reaction of both IgG1 and IgM. These facts are related to the Th1-polarised cytokine response and high *N. caninum* positivity identified in dams, and pups immunised with this formulation. These findings suggest a productive infection after challenge, with the reaction of IgM-like to dams of the positive control group. This fact suggests that the sequential administration of the peptides at a low dose in mice could induce a low rate of somatic hypermutation and clonal amplification due to the low T and B cell priming. In this context, the second immunological signal was low due to the low immune signalisation triggered after sequential immunisation.

Contrasting the vertical transmission rates of 0 and 6% in pups from dams immunised with standard doses of peptides concerning previous assays that used crude lysate of tachyzoites NC-Nowra for immunisation and NC-Liverpool for challenge in mice, this anterior assay showed a little reduction of transplacental transmission maintaining the rates of 53% and 63% in two experiments using dams of different ages (Miller et al. 2005). This difference could be explained by the priming of the immune response according to the differences in antigen processing and presentation; the pool of antigens from the crude lysate of tachyzoites could be less efficient during antigen processing in APCs. These antigens could be hydrolysed into small peptides in the phagosome of APCs, presenting only small, carefully folded antigens that could induce a nonspecific immune response (Ewanchuk and Yates 2018). Conversely, the standard dose peptides may be more efficient during antigen processing and presentation by APCs with little hydrolysis in the phagosome, thereby inducing an appropriate T-dependent immune response as mentioned above (Mayer and Impens 2021).

Similarly, the vertical transmission rate of 0% in pups from dams immunised with the standard dose of rsNcGRA1 peptide compares with the 63% rate observed in pups previously immunised with native recombinant NcGRA1 antigen (Ellis et al. 2008), and the chronic infection rate of 33% in dams immunised with the standard dose of rsNcSAG4 peptide compares with the 88.8% rate observed in dams previously immunised with native recombinant NcSAG4 antigen (Aguado-Martínez et al. 2009a), suggesting that native recombinant antigens failed to control vertical transmission in pups and chronic infection in dams. In contrast, the recombinant peptides derived from the immunogen motifs of the native antigens enhance the immunogenicity by decreasing the non-specific immune response with greater efficiency in processing and presenting antigens, as explained above. Additionally, the health indicators observed in the dams and pups that were immunised with the peptides in combination at standard doses align with previous findings that demonstrate the best vaccine effectiveness of antigen combinations (Aguado-Martínez et al. 2019). In this study, the peptides given in combination at the standard dosage effectively control the chronic infection in dams and prevent vertical transmission to their offspring.

Previous tests in mice using positive controls challenged with virulent strains resulted in over 80% postnatal mortality and vertical transmission, exceeding the capacity of the immune system to control the infection and showing unpromising effects of vaccine candidates (Aguado-Martínez et al. 2019). However, the parameters of positive control in this assay showed 31% overall mortality in pups, 70% infection in surviving pups, and 81% vertical transmission, aligned to parameters identified with lowest virulent strains; however, these strains generally showed the lowest vertical transmission rates and low positivity in surviving pups, contrary to those identified in this assay (Regidor-Cerrillo et al. 2010). Similarly, the brain lesions observed in pups of positive control aligned with findings reported after infection in BALB/c mice using tachyzoites with the lowest rounds of replication in cell culture (López-Pérez et al. 2008). However, this assay contrasts with studies that used virulent NC strains with the lowest round of replication in culture cells (Arranz-Solís et al. 2015), allowing a suitable challenge with the establishment of a chronic infection in pregnant dams associated with high vertical transmission capacity that permitted the analysis of the infection in pups, like challenges that used moderate virulent strains in mice (Regidor-Cerrillo et al. 2010). Finally, the immunological protection and health parameters observed in groups of standard dose-immunised confirm a consistent evaluation of peptide formulations in a mouse model.

## Conclusion

This study shows that recombinant rsNcGRA1 and rsNcSAG4 produced in *K. phaffii* administered alone and in combination at a standard dose reveal immunogenicity features, with the highest titres of antibodies with the predominance of IgG1, a balance between Th1 and Th2 cytokines and the ability to reduce the chronic infection in dams and a decrease of vertical transmission. The experimental model using pregnant BALB/c mice submitted to 2 × 10^5^ NC-1 tachyzoites with moderate virulence showed injury in females from the control group, in contrast to the health status identified in immunised dams with peptides in combination and rsNcGRA1 alone at a standard dose. Therefore, these results suggest a potential use of new-generation peptides as antigens in vaccine formulation against neosporosis.

## Data Availability

The authors confirm that the employed data supported the published claims, and the datasets analysed during the study are available with the corresponding author by reasonable request.
